# Informing the 2011 UN Session on Noncommunicable Diseases: Applying Lessons from the AIDS Response

**DOI:** 10.1371/journal.pmed.1001086

**Published:** 2011-09-06

**Authors:** Peter Lamptey, Michael Merson, Peter Piot, K. Srinath Reddy, Rebecca Dirks

**Affiliations:** 1 FHI 360, Public Health Programs, Arlington, Virginia, United States of America; 2 Duke Global Health Institute, Duke University, Durham, North Carolina, United States of America; 3 London School of Hygiene and Tropical Medicine, London, United Kingdom; 4 Public Health Foundation of India, New Delhi, India; 5 FHI 360, Program Sciences, Arlington, Virginia, United States of America

## Abstract

In advance of the September 2011 UN Summit on non-communicable diseases (NCDs), Rebecca Dirks and colleagues identify lessons from the AIDS epidemic that can inform the response to the growing epidemic of NCDs.

**Summary Points**

- The September 2011 UN High-Level Meeting on Noncommunicable Diseases provides an opportunity for the international community and national stakeholders to raise awareness and launch an effective global response to noncommunicable diseases (NCDs).
- Valuable policy lessons have been learned in the control of AIDS that can help inform the global dialogue when designing a NCD response in developing countries.
- The AIDS response demonstrates successes in advocacy and resource mobilization, priority setting, coalition building, strong national and community leadership, strengthening of community health infrastructures, and health systems strengthening.
- Weaknesses of the AIDS response to avoid when building a NCD response include creation of stove-pipe vertical programs, ineffectiveness of prevention efforts, and inefficient and uncoordinated use of resources.
- The lessons learned in the global response to AIDS are relevant to the likely outcomes of the UN High-Level Meeting on NCDs: (1) improvement in advocacy and recognition of the NCD burden, (2) greater attention in national planning and resource allocation, (3) a longer-term investment of donors, and (4) greater emphasis on strengthening health systems.

## Introduction

The United Nations General Assembly Special Session (UNGASS) on HIV/AIDS in 2001 was a critical event that dramatically enhanced the global AIDS response. Ten years later, the September 2011 UN High-Level Meeting on Noncommunicable Disease Prevention and Control provides a similar opportunity for the international community and national stakeholders to raise awareness and launch an effective global response to noncommunicable diseases (NCDs). It is an opportunity that should not be missed as it will not likely occur again.

Infectious diseases continue to have a devastating impact on the health and development of low- and middle-income countries (LMICs). However, NCDs have silently become “the poor world's greatest health problem” and the major causes of premature deaths in LMIC [1].

Despite the growing burden in developing countries, NCDs have received little attention and funding to date [2]. Tremendous advances in the control of NCDs were achieved in the second half of the 20th century, mostly to the benefit of wealthy countries. LMIC should not only look at the lessons learned in the control of NCDs in developed countries, but also those from other areas of public health, especially AIDS, which can inform the design of an effective and sustainable response to NCDs in developing countries.

## Applicable Lessons Learned for the NCD Response

### Strengths of the AIDS Response

First, the success in substantially increasing funding for AIDS programs over the last decade provides lessons in resource mobilization and advocacy to the NCD community. This dramatic increase was fueled by a variety of factors, including the impact of the disease on children and women, the availability of inexpensive diagnostics, reduced treatment costs, and the disparity in access to care between developed and developing countries. The AIDS response demonstrates the need to create a committed, diverse, and broad coalition comprised of multilateral and bilateral agencies, the donor community, national and regional leadership, and those whose lives are affected by the disease. Like AIDS, NCDs should be positioned as a leading cause of morbidity and mortality in LMIC with devastating social and economic impacts.

Next, policymakers must recognize that as devastating as the AIDS pandemic and other infectious diseases have been, the burden of NCDs have the potential to be much worse in LMICs. There is an urgent need to change national and international priorities and resource allocation policies to address these deficiencies. For example, Sridhar et al. propose the appointment of an NCD czar and leveraging existing high level leadership such as the UN Secretary General, Director General of World Health Organization (WHO), and the NCD Alliance to elevate and strengthen the leadership for NCD [3].

Another valuable lesson from the AIDS response is the role of strong national and community leadership. Such leadership was critical in achieving a coordinated and broad-based national response to AIDS over the course of many years. To tackle NCDs, the response must start with national leadership of country-owned and -managed strategies and programs. Just as national AIDS strategies have served as the basis for international support for AIDS funding, international and donor communities should invest in country-led NCD plans.

Furthermore, the AIDS response demonstrated that in order to develop sustainable and effective programs, community health infrastructures must be strengthened. Community-based organizations have successfully reached the most-at-risk populations, and experience from AIDS treatment points to the critical role of community-based care as part of a package of essential entitlements for access to healthcare [4]. The same must be done for NCD prevention and treatment.

Lastly, the AIDS response has shown that a primarily vertical health infrastructure can result in improvements in the laboratory infrastructure, pharmacy, and supply chain management of drugs and other medical commodities. Some of these health systems improvements have benefited non-HIV services, such as reproductive health [5]. The NCD response should use the results of vertical AIDS programming such as: (1) a valuable health services platform on which a more integrated and horizontal response can be built for other chronic diseases, (2) lessons learned in governance, resource mobilization, human rights approach and community mobilization, and an enhanced role of civil society, and (3) expertise in how to deliver effective and rapid health care in resource-poor settings such as program scale-up, program outreach, and chronic disease management.

### Weaknesses of the AIDS Response

In addition to the successes, the response to the AIDS epidemic provides lessons as to approaches that should be avoided when building a NCD response. To begin, the emergency nature of the AIDS response and the subsequent significant increase in funding resulted in the creation of many vertical programs. While AIDS programs have strengthened related aspects of health services, they have also fueled competition between AIDS and other health programs, hampering efforts in some countries to improve the capacity of the health system to address other priority health issues. These constraints are apparent across each of the WHO's six key health-systems' components [6] and have become more evident as development partners have accelerated efforts to respond to tuberculosis, malaria, and vaccine-preventable diseases. These disparities include: (1) distortion of resource allocation for national health priorities, (2) imbalance in access and quality of health services, (3) disparities in workforce compensation, (4) poor coordination and collaboration within the health sector, and (5) creation of parallel structures such as supply chain management, health care financing, and monitoring and evaluation programs.

While leveraging the improvements in health systems due to AIDS programs, the NCD response should avoid the weaknesses of a vertical response and develop a diagonal or horizontal approach to address a wide range of health conditions. There is in fact increased momentum among funders, such as the US President's Emergency Plan for AIDS Relief (PEPFAR) and The Global Fund for AIDS Tuberculosis and Malaria (GFATM), to strengthen national health systems to address a variety of health issues.

Second, despite some successes in AIDS prevention efforts, twice as many people are newly infected as are put on treatment. If we cannot treat ourselves out of the AIDS pandemic, it is equally unlikely that we would be able to treat ourselves out of a much larger NCD pandemic. An effective NCD response must ensure that adequate resources are allocated to comprehensive evidence-based prevention efforts.

Third, the complexity of AIDS prevention was not fully appreciated early on. It took many years for the AIDS response to evolve from a limited infectious disease approach to a broader public health and developmental response. We should not be similarly naïve when it comes to NCDs; the multisectoral dimensions of NCDs must be tackled from the onset.

Fourth, the urgent nature of the AIDS response and the need to rapidly implement interventions created inefficiencies and ineffective use of resources. Coordination among donors is still inadequate, limiting the ability to maximize and integrate the use of resources. The upcoming UN High-Level meeting should allow national governments and donors to synergize their efforts against NCDs.

Fifth, almost 30 years into the AIDS pandemic, we are still debating which of the many interventions are most effective. We need to agree on priority and evidence-based NCD interventions that are appropriate for local needs. The focus of the high-level UN meeting in 2011 on the four key NCDs (cardiovascular disease, cancer, diabetes, and chronic respiratory diseases) and their key risk factors (tobacco use, unhealthy diet, physical inactivity, and harmful use of alcohol) is a step in the right direction. Prevention of chronic infections such as human papillomavirus, hepatitis B virus, and hepatitis C virus, and prevention of cook stove smoke are additional NCD risk factors to consider in some settings.

Finally, the reduction in HIV treatment costs was realized late and the long-term costs of initiating and maintaining an increasing number of patients on antiretroviral treatment pose a serious challenge. This challenge is further compounded by the “treatment for prevention” debate that could result in millions of people with HIV infections being treated early and for many years. In this era of scarce resources, we need to ensure access to the most feasible, cost-effective, and sustainable NCD interventions to the populations at most risk.

## Conclusion

Noncommunicable diseases are no longer limited to industrialized countries. Many LMIC have completed their epidemiological transition with accelerated growth of NCDs, but without transitioning beyond diseases of the poor. NCDs are on the decline in high-income countries but rising rapidly in LMIC. By 2030, these chronic diseases will likely account for over two-thirds of deaths worldwide.

The upcoming UN High-Level Meeting on NCDs should lead to: (1) a significant improvement in advocacy and recognition of the NCD burden at the national and global levels, (2) greater attention in national planning and resource allocation, (3) modest immediate resource commitment, but more likely a longer-term investment of donors, and (4) greater emphasis on strengthening health systems for a more horizontal response to broader disease burdens. As we have indicated, several lessons from the AIDS epidemic can inform this meeting (Box 1).

**Box 1. Lessons for Global NCD Response**

- Position NCDs as a leading cause of morbidity and mortality in LMICs with devastating social and economic impacts
- Appoint czar or other high-level leadership to increase profile of NCDs
- Invest international and donor support in country-led NCD plans
- Strengthen community health infrastructures
- Adopt horizontal or diagonal health infrastructure to address multiple health issues simultaneously
- Focus on prevention, not just treatment
- Synergize donor and governmental efforts against NCD
- Agree on priority conditions, risk factors, and evidence-based interventions for NCD
- Ensure access to the most feasible, cost-effective, and sustainable NCD interventions to the populations at most risk

The global response to AIDS has demonstrated it is feasible to scale-up both prevention and treatment programs and drastically reduce both morbidity and mortality. Scale-up of even a limited number of interventions can have an even more dramatic reduction on NCDs. The AIDS response has taught us that efforts to address a pandemic are incremental and can take time. We need to be both opportunistic and strategic to achieve an NCD response of significant magnitude. Unless the pandemics of NCDs are addressed now, the lives of those living in developing countries will be saved from communicable diseases only to be lost prematurely from noncommunicable diseases.

## Abbreviations

LMIC: low- and middle-income country
NCD: noncommunicable disease
